# Psychological perspectives on divine forgiveness: 3. Trait self-control is associated with well-being through seeking divine forgiveness

**DOI:** 10.3389/fpsyg.2024.1292537

**Published:** 2024-02-19

**Authors:** Heather M. Maranges, Frank D. Fincham

**Affiliations:** The Family Institute and Department of Human Development and Family Science, Florida State University, Tallahassee, FL, United States

**Keywords:** trait self-control, seeking divine forgiveness, well-being, mental health, psychological distress, religiosity

## Abstract

**Introduction:**

Although a majority of the world’s population believes in a Higher Power and subscribes to a religion in which divine forgiveness is emphasized, little work has been done to understand individual differences associated with seeking divine forgiveness.

**Methods:**

Building on work that suggests trait self-control facilitates well-being, the current study (*N* = 439, undergraduate students) applies structural equation modeling (SEM) to test whether believers higher (vs. lower) in trait self-control are more likely to seek divine forgiveness, and, in turn, have better psychological health.

**Results and discussion:**

We find that people higher in self-control engage in more divine forgiveness seeking (*b* = 0.16), and seeking divine forgiveness represents one of the pathways associated with psychological health (i.e., seeking is associated with higher well-being, *b* = 0.21, and lower distress, *b* = −0.07). Crucially, we operationalize both positive (well-being and flourishing) and negative (depression, anxiety, stress) aspects of psychological health and control for religiosity. These results suggest that for those who believe in God, seeking divine forgiveness may be one mechanism that links individuals’ self-control to good psychological health, and this is not merely an artifact of higher levels of religiosity. Limitations and future directions are discussed.

## Introduction

Most people around the world believe in a Higher Power and subscribe to one of the world’s primary religions (Rye et al., 2000; Pew Research Center, 2012; Wormald, 2015), which emphasize the importance of divine forgiveness (i.e., forgiveness by Higher Power, Supreme Being, or God). Nonetheless, the psychological science of divine forgiveness is underdeveloped relative to those of interpersonal and self-forgiveness (Fincham, 2022). The current work adds to an emerging literature on the psychological correlates of divine forgiveness (e.g., Fincham and Maranges, 2024a,b) by focusing on an active decision point in the process model of divine forgiveness—seeking divine forgiveness (Fincham and May, 2023).^1^

Prior work suggests that trait self-control is associated with both religiosity (McCullough and Willoughby, 2009) and psychological well-being (De Ridder et al., 2012). Experiences of divine forgiveness have also been linked to better mental health (e.g., lower levels of depression and anxiety symptomatology, and higher levels of well-being; Krause and Ellison, 2003; Chen et al., 2018; Fincham and May, 2020) and there is a growing body of research linking religiosity to flourishing and health (e.g., Green and Elliott, 2010; Maltby et al., 2010; Abu-Raiya et al., 2016; Van Cappellen et al., 2016; Cohen and Johnson, 2017) Nonetheless, no extant work has tested the possibility that the seeking of divine forgiveness is one mechanism by which believers higher in trait self-control experience better mental health. The current work tests this possibility.

### Experiencing and seeking divine forgiveness

Divine forgiveness features as an important aspect of people’s religious beliefs and experiences across religious traditions. Although it is difficult to estimate the proportion of believers across the major world religions who engage in divine forgiveness processes, it is well established that such forgiveness from God is central to Abrahamic religions (Judaism, Christianity, Islam) and forgiveness more generally is central to as Buddhism, Hinduism, and Janism (Rye et al., 2000). Given that Christian theology highlights the importance of God as forgiving and seeking forgiveness from God (Marty, 1998), it is unsurprising that, compared to a nonreligious group, Protestant and Catholics experience high levels of divine forgiveness (Toussaint and Williams, 2008).

The first process model of divine forgiveness outlines the psychological benchmarks and decision points in believers’ quest for divine forgiveness (Fincham and May, 2023). After engaging in some thought, word, or deed, and perceiving it as a wrongdoing, the person must` choose to seek divine forgiveness in order to experience it. Although prior work has examined the experience of divine forgiveness, seeking divine forgiveness has only been examined in one study. Fincham and Maranges (2024a,b) developed a measure of seeking divine forgiveness by adapting the Transgression Narrative Test of Forgivingness (TNTF, Berry et al., 2001). Respondents consider standardized narratives describing transgressions and rate the likelihood of seeking forgiveness from a higher power for committing each transgression. They found that seeking forgiveness predicts subsequent experience of divine forgiveness and begin to answer the question, What kind of person is more or less likely to seek forgiveness?. Specifically, they find that people who are lower in avoidant attachment to God are more likely to seek divine forgiveness than people who are higher in avoidance, hinting at the possibility that seeking divine forgiveness is potentially aversive or difficult for some people. If seeking divine forgiveness is aversive or difficult, there are likely other individual differences related to regulation and effort that make people more or less likely to seek divine forgiveness. Here we propose that trait self-control is an important individual difference in shaping a person’s likelihood of seeking divine forgiveness.

Still open is the question of whether deciding to seek divine forgiveness benefits mental health. Prior work suggests that religious processes bolster well-being (e.g., Green and Elliott, 2010; Maltby et al., 2010; Abu-Raiya et al., 2016; Van Cappellen et al., 2016; Cohen and Johnson, 2017). For example, people who identified as religious were happier and healthier than people who did not, and this held above and beyond marital and job satisfaction (Green and Elliott, 2010). Such effects may be due in part to positive emotions (e.g., Van Cappellen et al., 2016) or meditative prayer (Maltby et al., 2010) elicited by religious practices. But they may also be linked to seeking divine forgiveness.

Although experiencing divine forgiveness has benefits for mental health (e.g., Krause and Ellison, 2003; Chen et al., 2018; Fincham and May, 2020), it remains to be determined whether seeking divine forgiveness is associated with better psychological health outcomes. Research on human forgiveness suggests that even just imagining seeking forgiveness from another person predicts lowered levels of negative affect (e.g., sadness, anger) and moral emotions (e.g., shame) (Witvliet et al., 2002) and cardiac stress responses (da Silva et al., 2017). Seeking divine forgiveness may also be associated with lower levels of markers of psychological distress. However, mental health is not merely the absence of psychological distress, and it is therefore important to assess positive aspects of mental health (e.g., well-being). Accordingly, the present research takes care to operationalize not only the negative (i.e., depression, stress, anxiety), but also the positive (i.e., well-being, flourishing) aspects of mental health.

### Trait self-control

Trait self-control is the ability to regulate one’s thoughts, feelings, and behaviors in line with one’s goals and with salient norms and standards (Baumeister et al., 2007). Theory and empirical work suggest that religiosity and self-control tend to positively covary (McCullough and Willoughby, 2009). Religious beliefs may motivate people to regulate themselves in accord with certain mores and moral rules and by potential rewards or punishments meted out in their current or anticipated afterlife. Important to consider, though, is the role that trait self-control can play day to day in facilitating engagement in religion-motivated psychological and social processes.

Seeking divine forgiveness may be difficult and effortful. People high in avoidant attachment to God are less likely to seek divine forgiveness, hinting that it may be an aversive step in the process of obtaining divine forgiveness (Fincham and Maranges, 2024a,b). People experience self-forgiveness as difficult and requiring effort (Fisher and Exline, 2006) and the self-forgiveness process begins with seeking such forgiveness (Exline et al., 2011). To the extent that self- and divine forgiveness depend on similar processes, it is reasonable to think that seeking divine forgiveness is difficult and/or requires effort and should then be associated with self-control.

The human forgiveness literature suggests that self-control facilitates forgiveness and, in turn, relationship maintenance. People with higher self-control invest more in maintaining their relationships with human partners (e.g., Vohs et al., 2011; Visserman et al., 2017) and tend to engage in more interpersonal (e.g., Burnette et al., 2014; Ghorbani et al., 2017; Ho et al., 2023) and self-forgiveness (e.g., Ghorbani et al., 2017; Hsu, 2021) processes. Similarly, people better able to emotion regulate are better able to forgive themselves (Hodgson and Wertheim, 2007). In turn, interpersonal (Ho et al., 2023) and self-forgiveness (Pelucchi et al., 2013) are positively associated with interpersonal relationship quality. These associations bear out longitudinally, with recent work demonstrating that trait self-control predicts later interpersonal forgiveness, which subsequently was associated with better relationship outcomes (i.e., satisfaction, commitment, closeness) (Ho et al., 2023).

Recall that other experimental data suggest that even the imagining of seeking interpersonal forgiveness predicts decreases in negative affect (e.g., sadness, anger) and moral emotions (e.g., shame) (Witvliet et al., 2002) and cardiac stress responses (da Silva et al., 2017). To the extent that self-forgiveness, interpersonal forgiveness, and divine forgiveness rely on similar socioemotional processes, we might assume that like human forgiveness, divine forgiveness, which begins with seeking, is effortful and preceded by (or at least associated with) self-control and predicts (or is at least associated with) well-being. Trait self-control has also been linked to higher well-being (e.g., lower depressed mood, higher happiness) longitudinally (Li et al., 2022), and the relationship between self-control and well-being emerged as significant with a medium effect size (*r* = 0.32) in a meta-analysis (De Ridder et al., 2012). For people who believe in a higher power, one potential mechanism by which trait self-control may confer mental health benefits is seeking divine forgiveness.

## The current work

The current work investigates whether people with higher, versus lower, self-control are more likely to seek divine forgiveness and, in turn, to experience better psychological health. We test this model in a population that tends to be healthy and Christian as an initial test of this model to reduce confounds (such as religious tradition or clinically compromised self-control). We operationalize seeking divine forgiveness and mental health in nuanced ways and employ structural equation modeling (SEM). We not only capture likelihood of seeking divine forgiveness with a standardized set of transgressions (Fincham and Maranges, 2024a,b), but also with each individual’s own personal transgression. Crucially, we also statistically account for religiosity, which is related to self-control and divine forgiveness. That is, by controlling for religiosity, we can more appropriately make the claim that self-control is associated with seeking divine forgiveness and, in turn, psychological well-being, rather than that these associations are due to the third variable of religiosity. Although the psychological processes associated with divine forgiveness may be particular to religious people, individuals differ in the extent to which they seek out and experience divine forgiveness (e.g., Fincham and Maranges, 2024a,b). Our primary hypothesis has to do with our overall model:

Main Hypothesis: Trait self-control will be associated with psychological distress (negatively) and psychological well-being (positively), and these links will be mediated through seeking divine forgiveness.

This model contains numerous hypotheses about bivariate associations:

Hypothesis 1: Trait self-control will be positively associated with seeking divine forgiveness.

Hypothesis 2: Trait self-control will be negatively associated with psychological distress.

Hypothesis 3: Trait self-control will be positively associated with psychological well-being.

Hypothesis 4: Seeking divine forgiveness will be negatively associated with psychological distress.

Hypothesis 5: Seeking divine forgiveness will be positively associated with psychological well-being.

### Method

#### Participants

Participants were 439 undergraduate students at a large southeastern U.S. public university taking courses in social sciences. Participation in the study was one option for obtaining a small amount of extra class credit. Only students who indicated a belief in a higher power were studied [i.e., we excluded participants who responded “no” to the question *Do you believe in a supernatural agent(s)* (e.g.*, God, Gods, a higher power*)*?*]. Participants ranged from 18 to 46 years old (*M_age_* = 19.91, *SD* = 1.81). See Table 1 for demographics. Four hundred and seventeen participants provided complete data, which allowed for more than 90% power to detect small to medium effects (e.g., based on prior work, Fincham and May, 2020, 2022; Soper, 2023). Soper (2023) calculates power by applying formulas for computing error function, lower bound sample size for a structural equation model, and normal distribution cumulative distribution function (CDF) (see Supplementary materials; Cohen, 1988; Westland, 2010).

**Table 1:** Sample sociodemographic frequencies.

**Race/Ethnicity**	***N***
White	296
Latina/o	60
African American or Black	46
Mixed Race	19
Asian	10
American Indian or Alaska Native	2
Native Hawaiian or Pacific Islander	1
Middle Eastern	1
Preferred not to say	4
**Religion**	***N***
Christian (Protestant, Catholic, etc.)	353
Jewish	19
Jewish and Catholic	2
Muslim	2
Muslim and Christian	1
Buddhist	2
Traditional/Native African Spirituality	1
Hindu	1
Hindu and Christian	1
Latter Day Saint	1
Did not identify with any specific religion	56

#### Procedure

Participants provided their demographics and responded to measures of trait self-control, seeking divine forgiveness, and negative and positive aspects of psychological health as part of a larger survey online. Participants also responded to a measure of religiosity, for which we statistically controlled. Thus, the study has a cross-sectional design. No data were collected before the study protocol was approved by the Florida State University Institution Review Board.

#### Materials

##### Trait self-control

Participants responded to the 13-item Self-Control Scale in its short form (Tangney et al., 2004). Respondents reported the extent to which they agreed with items such as *People would say that I have iron self-discipline* and *I am lazy* (reversed) on a 1 (*strongly disagree*) to 5 (*strongly agree*) scale. Scores were calculated by averaging across the items after reverse coding as needed (*M* = 3.37, *SD* = 0.63, α = 0.82).

##### Seeking divine forgiveness

To capture real and hypothetical divine forgiveness seeking via a latent factor, we measured two constituent variables.

The first measure is an adapted version of the Transgression Narrative Test of Forgivingness (TNTF) that captures the likelihood that people would seek forgiveness from a higher power given specific transgressions (Fincham and Maranges, 2024a,b). Participants responded to 5 narratives with the likelihood that they would seek forgiveness from God using a slider spanning from 0 to 100 with the prompt *Imagine yourself in such a situation and indicate how likely you are to seek God’s forgiveness for [summary of scenario]*. For example, participants reflected on cases in which they fail to mail a friend’s job application on time as promised, gossip about a coworker, and humiliate a family member. Narratives are provided in Supplementary materials. Scores were averaged across the 5 scenarios (*M* = 62.98, *SD* = 29.22, α = 0.93). This variable is denoted by “Seeking DF” in tables and figures.

For the second measure of seeking divine forgiveness, after reporting a transgression they committed against another person within the last month, participants responded to 2 items with the prompt *In regards to the hurt you wrote about to what extent have you... Asked a higher power (*e.g.*, God) to forgive you* and A*pologized to a higher power (*e.g.*, God) for your behavior.* Answers were indicated on a 1 (*not at all*) to 7 (*extensively*) scale. These two items were averaged to create a second measure of seeking divine forgiveness (*M* = 3.40, *SD* = 2.14, α = 0.96). This variable is denoted by “Transgression SDF” in tables and figures.

##### Psychological health

We operationalized psychological wellness both in terms of negative (depression, anxiety, stress) and positive indicators (well-being, flourishing).

###### Depression, anxiety, and stress

Participants responded to the 21-item Depression, Anxiety, and Stress Scale (DASS, Lovibond and Lovibond, 1995; Antony et al., 1998), indicating to what extent each item applied to them in the last week on a 4-point scale from *never* to *almost always*. Each subscale was assessed with 7 items, and we averaged scores across the 7 items for each. The depression scale included items such as *I was unable to become enthusiastic about anything* (*M* = 1.54, *SD* = 0.55, α = 0.77). The anxiety scale included the example item *I was aware of the action of my heart in the absence of physical exertion (*e.g.*, sense of heart rate increase, heart missing a beat)* (*M* = 3.86, *SD* = 1.63, α = 0.77). The stress scale included the example item *I found it hard to wind down* (*M* = 3.71, SD = 1.91, α = 0.89).

###### Well-being and flourishing

Participants responded to the 5-item WHO Well-Being Index (World Health Organization, 1998; for review on use of this scale, see Topp et al., 2015) on a 6-point scale, from *none of the time* to *all of the time*. Specifically, participants reported how they felt in the last 2 weeks on items (e.g., *I have felt cheerful and in good spirits* and *I have felt active and vigorous*). Scores were averaged across the 5 items (*M* = 4.02, *SD* = 0.97, α = 0.89).

Participants also responded to the 8-item Flourishing Scale on a scale from 1 (*strongly disagree*) to 7 (*strongly agree*), with items such as *I lead a purposeful and meaningful life* and *I am engaged and interested in my daily activities* (Diener et al., 2010). Items were averaged (*M* = 5.65, *SD* = 1.09, α = 0.96).

##### Religiosity

Participants responded to 2 items assessing religiosity on an 8-point scale (see Pearce et al., 2017). The first item asked *How important is religion in your life?* with response options *not at all* to *extremely*. The second item asked*, In general, how often do you attend religious services or meetings?* with frequencies spanning from *never* to *about once a day*. We averaged the two items to create the control measure of religiosity (*M* = 4.36, *SD* = 1.90, α = 0.77).

#### Analytic strategy

Our primary approach to data analysis was structural equation modeling (SEM) using AMOS 29 (Arbuckle, 2023). We modeled the association between trait self-control and the divine forgiveness latent factor, which in turn is associated with the latent factor of negative well-being and the latent factor of positive well-being. Only complete data response sets were used (*N* = 417).

Before testing our full model, we assessed the estimates and model fit of the measurement model, which included all observed and latent factors employed (i.e., self-control, divine forgiveness seeking, negative and positive psychological health latent factors, and religiosity) and covariances among them. All observed variables loaded onto their respective latent variables significantly and with acceptable estimates, which are presented in Table 2. Notably, depression was a slightly stronger indicator of psychological distress than anxiety or stress. The model fit was also acceptable, χ^2^ = 47.07, *df* = 18, *p* < 0.001, CFI = 0.98, NFI = 0.96, RMSEA = 0.06. We also provide the bivariate correlations among measured variables.

**Table 2:** Measurement model factor estimates for latent variables.

**Latent variable**	**Observed variable/Factor**	**Estimate**
Divine forgiveness seeking	Seeking DF	0.72***
	Transgression SDF	0.61***
Psychological distress	Depression	0.84***
	Anxiety	0.80***
	Stress	0.81***
Positive well-being	Well-Being	0.76***
	Flourishing	0.56***

***p < 0.001. Estimates are standardized. Seeking DF, standardized measure of seeking divine forgiveness. Transgression SDF, personal transgression-based seeking divine forgiveness.

### Results

See Table 3 for correlations among measured variables. As the main test of our model, we assessed whether trait self-control was positively associated with seeking divine forgiveness, which in turn was associated with mental health, both lower levels of psychological distress and higher levels of positive well-being.

**Table 3:** Correlations among measured variables.

	1	2	3	4	5	6	7	8	9
1. Trait Self-Control		0.14^**^	0.05	−0.42^***^	−0.32^***^	−0.34^***^	0.25^***^	0.28^***^	0.12^*^
2. Seeking DF			0.43^***^	−0.19^***^	−0.06	−0.07	0.22^***^	0.17^***^	0.56^***^
3. Transgression SDF				−0.05	0.09^*^	0.03	0.19^***^	0.07	0.47^***^
4. Depression					0.63^***^	0.66^***^	−0.53^***^	−0.42^***^	−0.09
5. Anxiety						0.71^***^	−0.35^***^	−0.27^***^	−0.04
6. Stress							−0.37^***^	−0.21^***^	−0.03
7. Well-being								0.41^***^	0.23^***^
8. Flourishing									0.07
9. Religiosity									

***p < 0.001, **p < 0.01, *p < 0.05. Seeking DF, standardized measure of seeking divine forgiveness. Transgression SDF, personal transgression-based seeking divine forgiveness.

**Figure 1:**
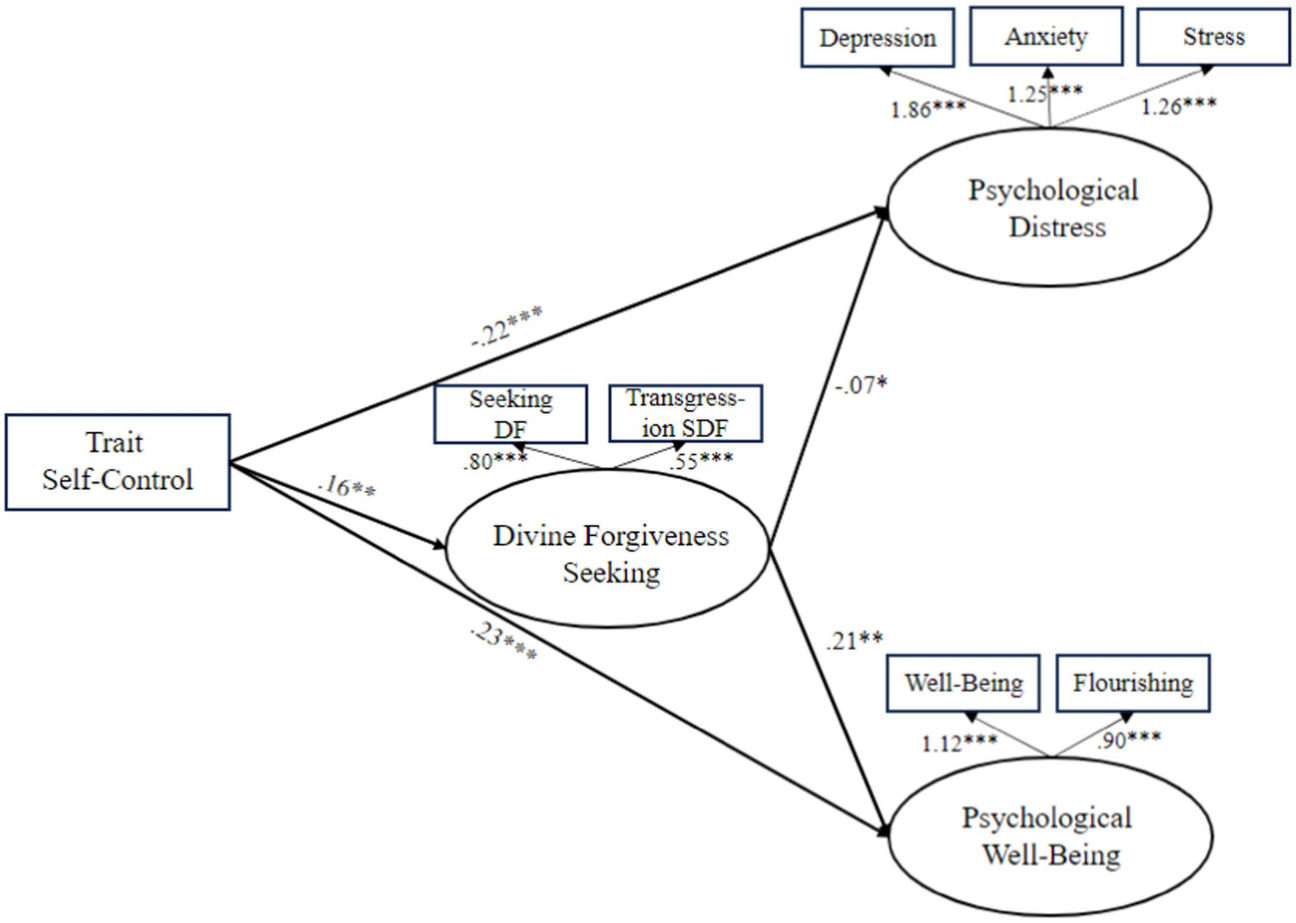
Structural equation model. ***p < 0.001, **p < 0.01, *p < 0.05. Error terms of observed variables modeled but not represented here. Estimates are standardized. Seeking DF, standardized measure of seeking divine forgiveness. Transgression SDF, personal transgression-based seeking divine forgiveness.

Next, we assessed whether trait self-control was associated with seeking divine forgiveness, which in turn was associated with psychological distress and positive well-being, via structural equation modeling (SEM). That is, we modeled both the direct effects of trait self-control on psychological distress and psychological well-being and the indirect effects of trait self-control on those psychological health outcomes through seeking divine forgiveness. Error terms of depression, anxiety, and stress were covaried, as were the error terms of well-being and flourishing given their measurement (i.e., because items are included in the same measure, as in the case of the DASS, or have shared method variance) and conceptual overlap (see Saris and Aalberts, 2003). As predicted, trait self-control was positively associated with seeking divine forgiveness, which was negatively associated with psychological distress and positively associated with psychological well-being (see Figure 1). The model fit was acceptable, χ^2^ = 37.42, *df* = 13, *p* < 0.001, CFI = 0.98, NFI = 0.96, RMSEA = 0.07. The astute reader will notice that the associations between the measured variables of depression, anxiety, and stress and the latent factor psychological distress as well as between the measured variable well-being and the latent factor well-being are greater than 1. Although these would be out of range if they were factor loadings, they are regression estimates. That they are greater than 1 does indicate, however, that the indicators of the latent variables are highly correlated and there may be multicollinearity (Jöreskog, 1999).

Indirect effects were assessed via 5,000-sample bootstrapping. There was partial mediation by seeking divine forgiveness: The indirect effect of self-control on psychological distress via seeking divine forgiveness was significant, *b* = −0.02, 95% CI [−0.05, −0.0001], *S.E.* = 0.01, *p* = 0.043, while the direct effect remained significant (represented in Figure 1). Likewise, the indirect effect of self-control on psychological well-being via seeking divine forgiveness was significant, *b* = 0.06, 95% CI [0.01, 0.14], *S.E.* = 0.03, *p* = 0.019, while the direct effect remained significant (represented in Figure 1). Note that these effects are small in size. The pattern of findings was similar when controlling for religiosity and religious denomination (i.e., Christianity vs. other vs. none) in two separate models (because they correlated significantly). Specifically, religiosity or religious denomination was included as a measured variable in two separate models (given they were highly correlated), and the associations between the control variable and both the psychological distress and psychological well-being latent factors were modeled. The only difference was that the link between seeking divine forgiveness and psychological distress became nonsignificant.

## Discussion

Many people profess a religious faith and believe in God (or a Supreme Being or Higher Power; Lundberg, 2010; Wormald, 2015), yet the psychological correlates of seeking divine forgiveness are not well understood. The current work built on work linking trait self-control to both religiosity and well-being to test whether divine forgiveness seeking mediates the association between trait self-control and positive mental health in believers. Employing novel measures of seeking divine forgiveness (e.g., Fincham and Maranges, 2024a,b), we found that people higher in trait self-control were more likely to seek divine forgiveness and, in turn, to experience less psychological distress (i.e., depression, anxiety, stress) and greater psychological well-being. Crucially, these results were not due to religiosity, for which we statistically controlled.

Fincham and May (2023) suggest that seeking divine forgiveness is a key component of the psychological process of divine forgiveness, but most prior work has focused on the experience of divine forgiveness and its links to mental health. The current work is the first to demonstrate associations between seeking divine forgiveness and both lower psychological distress and higher psychological well-being. This more active step of the psychological process of divine forgiveness may benefit mental health in a few different ways. It may be that, similar to work with human forgiveness (Witvliet et al., 2002; da Silva et al., 2017), seeking divine forgiveness works by alleviating negative feelings, such as sadness, anger, and shame, and physiological stress responses, decreasing psychological distress. When it comes to positive psychological well-being, seeking self-forgiveness may increase feelings of well-being by not only engendering positive effects that come from feeling forgiven (as in human forgiveness, Exline et al., 2011), but also by increasing feelings of closeness to and satisfaction with one’s relationship with God (as in human forgiveness, Ho et al., 2023).

The current results are also important for understanding the individual differences among believers that may be linked to the psychological processes associated with divine forgiveness. Results reported here suggest that not all people are equally likely to seek divine forgiveness when considering their own idiosyncratic transgressions as well as standardized transgressions. People higher in self-control are more likely to seek divine forgiveness than people lower in self-control. This makes sense given that seeking divine forgiveness is an important goal (i.e., to maintain one’s relationship with God and to not be sullied by one’s wrongdoings) but may be aversive and effortful to seek. People higher in self-control are better able to engage in the divine forgiveness seeking process to build toward those goals. This empirical insight is consistent with work finding that self-control facilitates relationship maintenance with other people (e.g., Visserman et al., 2017).

Past work has linked the experience of divine forgiveness to better mental health outcomes but has not as carefully considered the benefits of seeking divine forgiveness. The current work underscores that seeking divine forgiveness is associated with better mental health. Furthermore, it was found that seeking divine forgiveness benefits mental health both in terms of lower psychological distress and of higher psychological well-being. It may be that each step of psychological process involved in divine forgiveness provides some boost to mental health. Additional research is needed to test that idea.

Conclusions of the current work should be considered in light of several limitations. First, participants were mostly White women in college. Second, data were cross-sectional, though we theorize a temporal order of effects. Accordingly, findings should be replicated across time (i.e., trait self-control predicting later seeking of divine forgiveness predicting later well-being in a longitudinal design) and replicated in a more diverse (e.g., in gender, race, ethnicity, socioeconomic status) sample.

Notably, we focused on just one pathway: from self-control to seeking divine forgiveness to mental health. This is not to make the claim that trait self-control is a sole individual difference correlate of seeking divine forgiveness and therefore mental health. The extent to which an individual views procuring divine forgiveness as important to their authentic self, or goal strength may be another important factor (e.g., Stavrova et al., 2019). That is, believers who strongly prioritize the goal of maintaining their relationship with a Higher Power or being forgiven may shore up psychological resources to seek divine forgiveness. Furthermore, it may be that seeking divine forgiveness is associated with improved self-regulation over time, particularly when it comes to inhibiting problematic behaviors. Such an effect may have to do with the experience of divine forgiveness that comes after seeking it. Indeed, recent work suggests that the experience of divine forgiveness may benefit self-control (Maranges and Fincham, 2024a), but that work does not entail a longitudinal design. Relatedly, the current work does not account for the other steps in the process model of divine forgiveness, such as the experience of receiving divine forgiveness, which may be present and associated with the positive well-being correlates examined here.

The dynamic, temporal relationships among self-control, seeking divine forgiveness, and experiencing divine forgiveness should be investigated via systematic, longitudinal research. Such longitudinal research may also benefit from measuring objective behaviors of seeking divine forgiveness, as the current measures capture perceived likelihood—perhaps willingness or intentions—to seek divine forgiveness. By examining the relationships between the transgression-specific and likelihood of seeking divine forgiveness measures employed in the present study and objective behaviors of seeking divine forgiveness (e.g., asking God for forgiveness, conciliatory behaviors such as confession) as well as with psychological distress, future work may also shed light on why the two indicator measures of divine forgiveness were slightly differentially associated with indicators of psychological distress (i.e., depression, anxiety, stress). It may be that these measures differ in the intensity with which one desires divine forgiveness based on negative affect.

There are other psychological correlates of seeking divine forgiveness that have implications for mental health, including attachment to God and religious coping. Fincham and Maranges (2024a,b) found that avoidant attachment to God was associated with lower likelihood of seeking divine forgiveness, perhaps because it is effortful (per its association with self-control and past research). Interpersonal avoidant attachment has been linked to deficits in mental health, as evidenced by a recent meta-analysis (Zhang et al., 2022). Taken together, these findings implicate another possibility: that for avoidantly attached believers, not seeking divine forgiveness for wrongdoings is associated with poorer mental health.

Other work suggests that the experience of divine forgiveness links attachment to God to religious coping, especially positive religious coping (Maranges and Fincham, 2024b). It may be that people higher in self-control are not only seeking more divine forgiveness, but also engaging in more coping that directly supports positive well-being and buffers against psychological distress. Religious coping entails finding strength in one’s relationship with God and one’s religious community during times of stress and has been tied to psychological well-being in diverse populations (e.g., Scandrett and Mitchell, 2009; Park et al., 2018; Abu-Raiya et al., 2020). At the same time, one’s religion and relationship with God may be a source of struggle when facing stressors that make one doubt God’s love or fear Him (Exline et al., 2014), such as disasters (Zhang et al., 2021). During such times, communication with God (e.g., Wilt et al., 2023), and, from our perspective, seeking divine forgiveness especially, may be particularly difficult but important. Future research should test these possibility.

It is also important to consider that confounds other than religiosity may be at play and should therefore be taken into account by future research. For example, the extent to which individuals feel shame and guilt may be associated with self-control (e.g., Tangney et al., 2004), seeking divine forgiveness (see Fincham and May, 2022), and mental health (e.g., Pugh et al., 2015; Carden et al., 2020).

Prior work suggests that religiosity and spirituality are negatively associated with depressive symptoms and depression, especially individuals from high levels of adversity and suffering. Specifically, meta-analytic evidence suggests that religiosity and spirituality serve as a buffer against depression, especially for people who were experiencing high levels of stress due to recent life events (Smith et al., 2003) or who had psychiatric symptoms (Braam and Koenig, 2019). Although our results provide a heuristic proof of concept that self-control may be associated with better psychological health through divine forgiveness seeking, this should be tested in populations facing challenges of clinical depressive symptoms or adversity, for whom religious processes may be particularly important in serving as protective factor against psychological distress.

Finally, on a methodological note, our model did not assess constituent items of scales as indicators of latent factors. For example, an alternative approach might include all 21 items of the DASS as indicators of psychological distress. Such a bottom-up approach would provide insight on which specific items are the strongest indicators of the constructs, providing more fine-grained empirical results and less bias, but it would also require extremely high statistical power via large sample size (i.e., more than 4,000 participants, Soper, 2023). Instead, our approach captured constructs of interest with extant scales and subscales, which served as indicators, and afforded sufficient statistical power and model efficiency. For the pros and cons of these approaches, see Matsunaga (2008). Future research may benefit from employing a more bottom-up methodological approach in the study of self-control, divine forgiveness seeking, and mental health.

Notwithstanding the limitations of our study, the current work is of heuristic value as it is the first to provide insight on the mediating role of divine forgiveness seeking in linking trait self-control to better mental health. People higher in self-control are more likely to seek divine forgiveness than those lower in self-control, and, in turn, to experience more well-being and flourishing and less depression, anxiety, and stress. These results not only tell us about the type of person who is more likely to seek divine forgiveness—a highly controlled individual—but also tell us about divine forgiveness seeking itself—it is associated with self-control.

The current work also has implications for more applied work. Intervention studies aimed at increasing well-being of people who believe in a higher power may aim to increase self-control and, in turn, divine forgiveness seeking. Self-control training interventions effectively improve people’s self-control and outcomes such as health behaviors, physical health, and well-being (see meta-analysis, Beames et al., 2017). Such work should be extended to clinical populations to establish therapeutic efficacy. Thus, not only does this intervention approach test a novel theoretical pathway to well-being, but it does so by providing data from which causal conclusions can be made about the links between self-control and divine forgiveness as well as well-being.

## Data availability statement

The raw data supporting the conclusions of this article will be made available by the authors, without undue reservation.

## Ethics statement

The studies involving humans were approved by Florida State University Institutional Review Board. The studies were conducted in accordance with the local legislation and institutional requirements. The participants provided their written informed consent to participate in this study.

## Author contributions

HM: Conceptualization, Formal analysis, Investigation, Methodology, Visualization, Writing – original draft, Writing – review & editing. FF: Funding acquisition, Investigation, Writing – review & editing.

## Footnotes

^1^Here and throughout, we are not making ontological claims about divine forgiveness. Rather, our focus is on the psychology of seeking and/or experiencing divine forgiveness, which is an important psychological process for religious individuals.
