# Supplementary material for: Psychological perspectives on divine forgiveness: 3. Trait self-control is associated with well-being through seeking divine forgiveness
Source: Front Psychol. 2024 Feb 19;15:1292537. doi: 10.3389/fpsyg.2024.1292537 (PMC10909953; doi:10.3389/fpsyg.2024.1292537)
Supplement: Supplementary file 1 [file Data_Sheet_1.pdf]

Psychological perspectives on divine forgiveness:

3. Trait self-control is associated with well-being through seeking divine forgiveness

SUPPLEMENTAL MATERIALS

Formulas used by Soper (2023) Power Analysis for SEM Calculator

Soper, D. S. (2023). A-priori Sample Size Calculator for Structural Equation Models [Software]. Available from https://www.danielsoper.com/statcalc

Error function:

$$\operatorname{erf}(x) = \frac{2}{\sqrt{\pi}} \int_0^x e^{-t^2}\,dt.$$

Lower bound sample size for a structural equation model:

$n = \max(n_1, n_2)$

where:

$$n_1 = \left\lceil 50\left(\frac{j}{k}\right)^2 - 450\left(\frac{j}{k}\right) + 1100 \right\rceil$$

$$n_2 = \left\lceil \frac{1}{2H}\left(A\left(\frac{\pi}{6} - B + D\right) + H + \sqrt{\left(A\left(\frac{\pi}{6} - B + D\right) + H\right)^2 + 4AH\left(\frac{\pi}{6} + \sqrt{A} + 2B - C - 2D\right)}\right) \right\rceil$$

$$A = 1 - \rho^2$$

$$B = \rho \arcsin\left(\frac{\rho}{2}\right)$$

$$C = \rho \arcsin(\rho)$$

$$D = \frac{A}{\sqrt{3 - A}}$$

$$H = \left(\frac{\delta}{z_{1-\alpha/2} - z_{1-\beta}}\right)^2$$

where j is the number of observed variables, k is the number of latent variables, ρ is the estimated Gini correlation for a bivariate normal random vector, δ is the anticipated effect size, α is the Sidak-corrected Type I error rate, β is the Type II error rate, and z is a standard normal score.

Normal distribution cumulative distribution function (CDF):

$$F(x; \mu, \sigma^2) = \frac{1}{2}\left[1 + \operatorname{erf}\left(\frac{x - \mu}{\sigma\sqrt{2}}\right)\right],$$

where μ is the mean, σ is the standard deviation, and *erf* is the error function.

Seeking Divine Forgiveness Measure

Below are a number of situations. Your task is to imagine yourself behaving in the way described in each situation even though the behavior may not be something you would do. People respond in different ways to these situations in terms of seeking forgiveness. Then indicate how you think you would respond to the situation (do NOT indicate how you or others think you should respond):

1. You have a paper due at the end of the week when you run into someone who you occasionally see in the class. This person has already completed the paper for the class and you tell them that you feel under a lot of time pressure and ask them to lend you their paper for some ideas. They agree, and feeling under great duress you find yourself simply retyping the paper and handing it in. The professor recognizes the paper, calls both of you to her office, scolds the person who gave you the paper, and says they are lucky she doesn't put both of you on academic probation. Imagine yourself in such a situation and mark how likely you are to seek God's forgiveness for what you did. [0 to 100 slider]

2. You offer to drop off a job application for a friend at the post office by the deadline for submission. A week later, your friend gets a letter from the potential employer saying that his/her application could not be considered because it was postmarked after the deadline. You remember that on the way to the post office you had met an old friend, went to lunch, and lost track of time. When you remembered the package, it was close to closing time at the post office and you would have to have rushed frantically to get there; you had then decided that deadlines usually aren't that strictly enforced so you waited until the next morning to deliver the package. Imagine yourself in such a situation and indicate how likely you are to seek God's forgiveness for not delivering the application on time. [0 to 100 slider]

3. You just started a new job and it turns out that a classmate from high school works there, too. Even though the classmate wasn't part of your crowd, there's at least a face you recognize. You two hit it off right away and talk about old times. A few weeks later, you are having lunch in the cafeteria with several of your coworkers, and tell them about something your old classmate did back in school; they laugh at the story and one co-worker even makes a snide and hostile comment about your old classmate. The next day your old classmate tells you that she was nearby and overheard the conversation with the co-workers and that what you told them is something that she is deeply ashamed of and did not want anyone to know about. Imagine yourself in such a situation and mark how likely you are to ask seek God's forgiveness for telling others your old classmate's secret. [0 to 100 slider]

4. An acquaintance tells you about a job that he or she really hopes to be hired for. Without telling them, you then apply for the job and end up getting it. A couple of days later, the acquaintance tells you that they did not get the job and now will not be able to pay their rent. Imagine yourself in such a situation and mark how likely you are to seek God's forgiveness for what you did? [0 to 100 slider]

5. You accompany your family to a New Year's party. The atmosphere at the party is warm and friendly. You have a drink and are soon chatting with a group. You end up humiliating a family member by sharing a story about them that they did want anyone outside the family to know. The group laughs at the story; the family member turns red with embarrassment and then leaves the party. Imagine yourself in such a situation and mark how likely you are to seek God's forgiveness for hurting the family member.
[0 to 100 slider]

Fincham, F. D., and Maranges H. M. (2023). Psychological perspectives on divine forgiveness: Seeking divine forgiveness.

Exploratory analysis. Self-control has two components. Inhibitory self-control is the ability to resist an urge or avoid engaging in a thought, feeling, or behavior that is goal-inconsistent, whereas initiatory self-control is the ability to willfully engage in a goal-consistent thought, feeling, or behavior. Prior work suggests that inhibitory self-control and initiatory self-control may be differentially associated with behavior, such as smoking, drinking alcohol, exercising, and studying (de Ridder et al., 2011). Accordingly, we explore whether inhibitory and initiatory self-control are similarly or differentially associated with seeking divine forgiveness and, in turn, psychological distress and well-being. See Table 1S for subscale items (based on de Ridder et al., 2011).

The model fit was acceptable, $\chi^2 = 42.19$, $df = 17$, $p = .001$, CFI = .98, NFI = .97, RMSEA = .06. See Figure 1S. Neither initiatory ($p = .917$) nor inhibitory ($p = .097$) self-control were significantly associated with seeking divine forgiveness. Initiatory self-control was significantly negatively associated with psychological distress and positively associated with psychological well-being. Inhibitory self-control was significantly negatively associated with psychological distress but unrelated to psychological well-being. The mediation pathway from inhibitory self-control to psychological distress through seeking divine forgiveness was significant, $b = -.01$, 95% CI [-.05, -.0001], *S.E.* = .01, $p = .048$. Likewise, there was an indirect effect of inhibitory self-control on psychological well-being through seeking divine forgiveness, $b = .05$, 95% CI [.0001, .132], *S.E.* = .03, $p = .048$. There were no indirect effects of initiatory self-control on either psychological distress ($p = .936$) or psychological well-being ($p = .928$). We cannot rule out whether these results are due in part to limited reliability of the scales.

Table 1S

Inhibitory and Initiatory Self-Control Subscales

**Inhibitory Self-Control**	**Initiatory Self-Control**
I am good at resisting temptation.	I am lazy (R).
I have a hard time breaking bad habits (R).	I have trouble concentrating (R).
I do certain things that are bad for me, if they are fun (R).	I am able to work effectively toward long-term goals.
I refuse things that are bad for me.	I often act without thinking through all the alternatives (R).
Sometimes I can’t stop myself from doing something, even if I know it is wrong (R).	
Pleasure and fun sometimes keep me from getting work done. (R)	

Note. (R) indicates reverse coding.

Figure 1S

Structural equation model with initiatory and inhibitory self-control

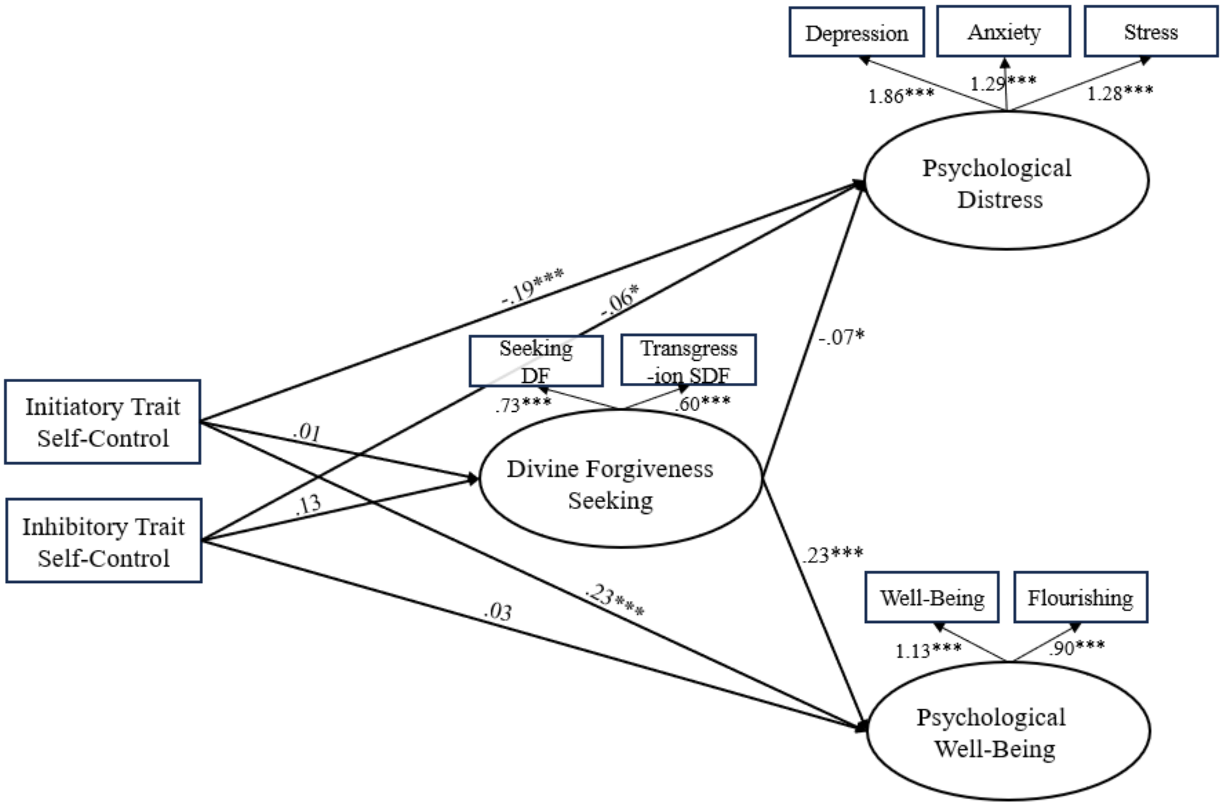

Note. ***p < .001, **p < .01, *p < .05. Error terms of observed variables modeled but not represented here. Estimates are standardized. Seeking DF = standardized measure of seeking divine forgiveness. Transgression SDF = personal transgression-based seeking divine forgiveness.
